# Women’s techniques for pleasure from anal touch: Results from a U.S. probability sample of women ages 18–93

**DOI:** 10.1371/journal.pone.0268785

**Published:** 2022-06-29

**Authors:** Devon J. Hensel, Christiana D. von Hippel, Charles C. Lapage, Robert H. Perkins

**Affiliations:** 1 Department of Pediatrics, Division of Adolescent Medicine, Indiana University School of Medicine, Indianapolis, Indiana, United States of America; 2 Department of Sociology, Indiana University Purdue University-Indianapolis, Indianapolis, Indiana, United States of America; 3 OMGYES Research Group, For Goodness Sake LLC, Berkeley, California, United States of America; University of Westminster, UNITED KINGDOM

## Abstract

The study purpose was to assess, in a U.S. probability sample of women, the specific ways women have discovered to experience pleasure from anal touch. Through qualitative pilot research with women that informed the development of the survey instrument used in this study, we identified three previously unnamed, but distinct, anal touch techniques that many women find pleasurable and that expand the anal sexual repertoire beyond the more commonly studied anal intercourse behaviors: Anal Surfacing, Anal Shallowing, and Anal Pairing. This study defines each technique and describes its prevalence among U.S. adult women. Weighted frequencies were drawn from the Second OMGYES Pleasure Report—a cross-sectional, online, national probability survey of 3017 American women’s (age 18–93) sexual experiences and discoveries. Participants were recruited via the Ipsos KnowledgePanel^®^. Data suggest that 40% of women find ‘Anal Surfacing’ pleasurable: sexual touch by a finger, penis, or sex toy on and around the anus. Approximately 35% of women have experienced pleasure using ‘Anal Shallowing’: penetrative touch by a finger, penis, or sex toy just inside the anal opening, no deeper than a fingertip/knuckle. Finally, 40% of women make other forms of sexual touch more pleasurable using ‘Anal Pairing’: touch on or inside the anus that happens at the same time as other kinds of sexual touch such as vaginal penetration or clitoral touching. These data provide techniques that women can and do use to explore the anus as a pleasurable region for touch—which can enable women to better identify their own preferences, communicate about them and advocate for their sexual pleasure.

## Introduction

The purpose of this study is to use nationally representative data to describe the ways in which adult women use different anal touch and stimulation techniques–defined for the purposes of this paper as penetration or internal/external stimulation of the anus with a penis, finger(s), lips/tongue, sex toy, or other object [1]–as a means of achieving sexual pleasure. The geography of the anus makes it a highly pleasurable sex organ: it contains a dense network of sensory nerves that participate with the genitals in the engorgement, muscular tension and contractions of sexual arousal and orgasm [2, 3].

Despite this anatomic pleasure potential [4], much of the existing published scientific research on anal sexuality narrowly focuses on *what* body part–typically a penis, and less often a mouth/tongue or finger–is stimulating or penetrating the anus, rather than thinking more broadly about *how* women and their partners may individuate stimulation and penetration techniques. For example, existing studies most commonly operationalize “anal sex” for women in terms of penetration of their anus with a male partner’s penis. The prevalence of women’s participation in anal sex varies depending both upon the behavior as well as the timeframe of reference. Different nationally representative studies from the past decade suggest that about a third of women have lifetime experience with penile-anal sex [5–10]. Between 11.8% and 13.2% have engaged in penile-anal sex in the past year [5, 6] and about 10% have done so in the past 90 days [11]. Less than five percent of women report engaging in anal sex during their most recent sexual experience [12]. A less developed body of literature using nationally representative data from the United States and from Australia suggests that women have also participated in other anal stimulation and penetration techniques, such as digital-penetration, manual stimulation, and/or oral-anal contact. This work suggests that between five percent and 43% of women have participated in oral-anal contact [1, 13, 14], between 15% and 20% have had a partner digitally stimulate and/or penetrate their anus [1, 15] and very few have ever had a partner insert a fist into their anus [14]. None of this literature assessed the specific touch techniques that were used by women and their partners to stimulate the anus internally or externally, or to what extent they were pleasurable.

A lack of detailed scientific studies on women’s anal pleasure techniques means that women and their partners likely receive more information from adverse-outcome focused peer-reviewed literature or from print/electronic/film media, about what women’s anal sex lives “look” like and/or how they “should” feel about engaging specific practices. In public health literature, for example, anal sex–especially when defined as women’s receipt of penile penetration of their anus–typically broadly quantifies frequency and with whom anal sex practices occur, often framing the outcomes in terms of potential “risks” (e.g. sexually transmitted infections, pain, bodily injury) [16], rather than emphasizing how women and partners work to choose specific stimulation and penetration techniques that are associated with positive outcomes like pleasure. Peer-reviewed scientific studies do examine women’s experiences with receptive penile-anal sex, and while some do document pleasure as a motivator [17, 18], far more emphasize adverse aspects, such as the cultural stigma associated with anal sex, or how women do not enjoy or are coerced into participating in anal sex [19]. Next to nothing is known about either the ways in which women actively choose different techniques that are not penile-anal penetration or about how pleasure levels may vary across these techniques [20]. Popular print and electronic media interest in anal sex has increased in the past decade, with common emphasis on the “right way” or “how to”–often using slang or ambiguous terms (e.g. “butt sex”)–to both prepare for and to engage in penile-anal sex [21, 22]. These sources rarely acknowledge a wider repertoire of anal stimulation and stimulation methods that women can and do choose [23].

In the current paper, we use data from the *second OMGYES Pleasure Report* to address both a measurement and a sampling gap in the literature about women’s experiences with anal stimulation and penetration. The *second OMGYES Pleasure Report* is a nationally representative study designed to investigate women’s preferences for genital touch/stimulation and penetration, including those involving the anus. From a *measurement* perspective, it is important for sexual pleasure research to assess a range of *ways* that women use for anal touch that are not currently explored in existing studies, as it elicits the information needed to both validate what women are doing, and also to provide the techniques names that are accessible and non-ambiguous. Usable terminology scaffolds a woman’s ability to communicate with partners, as well her ability to learn new techniques and/or to tinker with existing techniques she is already using [24]. We have used this level of specificity in detail and naming in a prior nationally representative study, the *first OMGYES pleasure report*, which explored women’s experiences with external vulvar and clitoral touch techniques [25] as well as a previous paper using data from the *second OMGYES Pleasure Report* describing women’s preferences for internal vaginal touch techniques [24]. In both these previous studies, rather than asking participants whether they liked to be touched externally or inside their vagina, the data demonstrated that women use different *ways* of touching–different locations, pressure, shapes, and patterns–as a means of increasing their sexual enjoyment. From a *sampling* perspective, although existing nationally representative studies in the United States assess women’s participation in penile-anal sex [5–12], the lack of other anal stimulation and penetration techniques means that their prevalence and patterns at the population level are unknown [24]. Such data are important to be able to reflect the experiences of all women in the United States.

Accordingly, the purpose of the current study was to use nationally representative probability data from *the second OMGYES Pleasure Report* to investigate women’s experiences and preferences for anal stimulation and penetration.

## Materials and methods

### Data collection

Data for the current study were drawn from the *second OMGYES Pleasure Report*—a cross-sectional, online, nationally representative survey of sexual behaviors, sexual attitudes, relationships, sexual satisfaction, and experiences with genital touching among women aged 18 and over in the United States. The study was conducted in July 2018 by Ipsos Research using their KnowledgePanel^®^ (Menlo Park, California) to recruit a probability-based web panel designed to be representative–including an oversample of lesbian and bisexual women–of all noninstitutionalized U.S. women. The 90-item online survey took a median of 29 minutes to complete, was available in English, and was open for participation from July 12-July 31, 2018. Questions assessed participation in demographics, sexual behavior background, as well as lifetime participation in different types of **Anal Surfacing, Anal Shallowing, and Anal Pairing**. All sexual touch technique items are original to this study and have not been yet examined in the peer-reviewed literature. Definitions and sexually explicit line drawing illustrations of these four techniques are provided in an online S1 Table. Images contained in this table are visually graphic.

Of those who opened the study link, 88.8% (3017/3398) completed the survey (49.7% [3017/6123] of the initial sampling frame) and represent the analytical sample in this study. This completion rate is similar to other Ipsos-conducted nationally representative studies of sexuality and sexual behavior (44%–51%) [5, 26–28]. Post-stratification, study-specific weights adjusted for over- or under-sampling as well as non-response. Participants provided electronic informed consent. All study procedures were approved by the institutional review board at Indiana University School of Medicine (IRB # 1801846511). Additional methodological details–for both this survey as well as the development of this survey–are available in Hensel et al. [24] All data used in this study are available through the ICPSR open data sharing consortium [29].

### Measures

Participants were asked about their experiences with three domains of anal touch and stimulation techniques, including **Anal Surfacing, Anal Shallowing**, and **Anal Pairing**. These domains were originally identified as part of qualitative work conducted prior to the larger study (additional detail on this work is available in Hensel et al., 2021: [24]). Anal Surfacing emerged through a theme of many women’s discovery that anal touch could be pleasurable when the anus was reconceptualized as a flat erogenous zone, which could be stimulated with touch on the surface, as opposed to solely as an opening for penetration. Anal Shallowing emerged from the qualitative analysis through a theme that demonstrated many women had discovered anal pleasure with shallow penetrative touch just inside the anal opening, in contrast to the deeper penetration commonly associated with the concept of anal sex. Anal Pairing emerged from the theme of women’s insight that when anal touch occurs simultaneously with other forms of sexual touch (e.g., vaginal penetration, clitoral touching), it can make the sexual experience more pleasurable. This is consistent with findings from our previous research on techniques women use for pleasure during vaginal penetration where Pairing, in this case of clitoral stimulation with vaginal stimulation, helped 69.7% of women orgasm more often or make vaginal penetration more pleasurable [24]. Definitions and sexually explicit line art illustrations of all three anal touch techniques are provided in the S1 Table.

Questions about anal touch were prefaced in the survey with a statement that said: “One area of sexuality that’s rarely talked about is anal touch/stimulation, even though nearly half of Americans have tried anal play. We are not talking just about anal penetration or what’s commonly called ‘anal sex.’ Rather, we are talking about any kind of touch of the outside or inside of the anus or butthole with a fingertip, toy, penis or anything else.”

#### Anal surfacing

Participants were first asked the extent to which they had ever found different methods of “touch on the outside of your anus/butthole” pleasurable when used either during sex with a partner or alone during masturbation (all: four-point Likert scale: 1. not pleasurable to 4. very pleasurable; or “don’t know or never tried”). The four items related to specific sub-forms of Anal Surfacing were: “with your own finger,” “with your partner’s finger,” “with a sex toy,” and “with a penis.” All items were dichotomized as pleasurable (a little/somewhat/very pleasurable) vs. not (not at all pleasurable/don’t know or never tried) for analysis. A response of a little/somewhat/very pleasurable to at least one anal surfacing item indicated a woman had found any form of anal surfacing pleasurable.

#### Anal penetration and shallowing

Participants were asked the extent to which they had ever found different methods of “penetration inside of your anus/butthole (either shallow or deeper)” pleasurable when used either during sex with a partner or alone during masturbation (all: four-point Likert scale: 1. not pleasurable to 4. very pleasurable; or “don’t know or never tried”). The four items related to specific sub-forms of Anal Penetration were: “with your own finger,” “with your partner’s finger,” “with a sex toy,” and “with a penis.” All items were dichotomized as pleasurable (a little/somewhat/very pleasurable) vs. not (not at all pleasurable/don’t know or never tried) for analysis. A response of a little/somewhat/very pleasurable to at least one anal penetration item indicated a woman had found any form of anal penetration pleasurable.

Women who found any form of anal penetration pleasurable were then asked the multiple response item “You mentioned you found penetration inside your anus pleasurable. At what depth have you found anal stimulation most pleasurable?” Participants selected the depth that applied from the following list: “just barely inside of anus/butthole (such as the very tip of a finger);” “about 1-knuckle inside;” “2-knuckles deep or as deep as an entire finger can go;” “deeper inside than an entire finger can usually reach;” or they selected “I don’t know.” A selection of either the “just barely inside of anus/butthole (such as the very tip of a finger)” or the “about 1-knuckle inside” depth response option indicated a woman had found any form of Anal Shallowing pleasurable. A selection of any of the greater depths of penetration as most pleasurable indicated a woman enjoyed deeper anal penetration.

#### Prior experience and pleasure from any form of anal touch

A response of a little/somewhat/very pleasurable to at least one Likert scaled anal surfacing or anal penetration (shallow or deeper) item indicated a woman had experienced pleasure with any form of anal touch. Conversely, a response of “I don’t know or never tried” to every Likert scaled anal surfacing and anal penetration (shallow or deeper) item indicated a woman had no prior experience with or knowledge of pleasure from any form of anal touch. Subsequent measures described below were asked only of the subgroup of women with prior experience of pleasure from any form of anal touch. These measures were used to evaluate patterns observed in the qualitative research phase relating to women’s experiences of discovering anal touch could be pleasurable and specific ways in which it was pleasurable for them.

#### Assessment of anal pleasure discovery patterns

To discern whether women’s enjoyment of anal touch was immediate or gradual, women who indicated that they found any form of external anal touch pleasurable were asked which statement best described their experience with touch outside their anus: either, “You found it pleasurable from the first time you tried it;” or, “You didn’t find it pleasurable at first, but learned to enjoy it over time.” In the same fashion, women who indicated they found any form of anal penetration pleasurable were asked which of the same two statements as above best described their experience with anal penetration.

Additionally, women who previously indicated they enjoyed any form of anal touch were asked to enter into number boxes the approximate ages in years at which they discovered that touch on the outside of the anus could be pleasurable and/or discovered that touch inside the anus could be pleasurable. If they did not recall their age, women entered “99;” all responses of “99” and women missing responses were excluded from the analyses of age at discovery. Additionally, age 14 years at discovery was used as the lower bound for analysis since this is a typical age at which young people in U.S. states can consent to sexual, reproductive health services without parental permission [30].

Factors contributing to women’s discovery of pleasure with any form of anal touch were assessed with a multiple response item: “Over time, which of the following do you think made you realize anal stimulation could be pleasurable for you?” Response options were “during self-exploration/masturbation, I found a way of anal stimulation that I liked;” “a partner approached anal sex in a way that worked for me;” “trying anal touch or anal sex on my own terms (e.g., communicating about the way I wanted to do it and having that respected);” “once I/we used enough lubricant;” “I had enough "warm up time" beforehand;” “trying it with someone I loved or deeply cared for;” “a partner just barely touched or brushed by my anus/butthole and I enjoyed it;” “I felt an increased desire for anal sex during or since pregnancy;” “I felt an increased desire for anal sex during or after menopause;” “other.”

#### Anal pairing and types of pleasure experienced from anal touch

A single multiple response item asked women about the ways in which anal touch is pleasurable for them: “Other women have suggested the following reasons why anal stimulation is pleasurable. What do you think it is about anal stimulation that makes it pleasurable for you?” Respondents could select one or more of the following response options to describe their experience: “I can have orgasms just from anal touch/stimulation;” “it can make my orgasms feel more intense;” “it can make it easier for me to have an orgasm during other kinds of touch;” “when it happens at the same time as other kinds of touch (like vaginal sex or clitoral touching), it can make the experience more pleasurable;” “It has its own unique sensation that I find pleasurable;” “I get a thrill from the feeling that anal play is taboo;” “it feels profoundly intimate and emotional;” the pleasure feels fuller or ‘bigger’ than other kinds of sexual pleasure;” “other.” The selection of one or both of the two response options relating to pleasure from anal stimulation as an enhancement of their pleasure when done simultaneously with other touch indicated a woman found Anal Pairing pleasurable.

### Statistical procedure

Weighted frequencies were calculated to assess the prevalence of women who have used Angling, Rocking, Shallowing, Pairing, and their sub-forms to make vaginal stimulation and penetration more pleasurable. We excluded from analysis of each item any participant whose response to that item was missing. IBM SPSS Statistics software was used for all analyses.

## Results

### Respondent characteristics

Weighted respondent demographic characteristics—including age, gender, race/ethnicity, education, household income, and geographic region of residence in the US, sexual orientation and relationship status—are presented in Table 1. Women ranged in age from 18 to 93 with a median age of 48 years. The majority of women self-described their sexual orientation as heterosexual (91.2%). Most women were in a married, committed, or dating relationship, with only 21.6% describing their relationship status as single and not dating at the time of the survey.

**Table 1:** Demographics of national probability sample of 3017 U.S. women (weighted).

Demographics	n	Percentage
**Sexual Orientation**		
Gay or Lesbian	68	2.2%
Bisexual	176	5.8%
Something else	21	0.7%
Straight/heterosexual	2752	91.2%
**Gender Identity**		
Cisgender	2973	98.5%
Transgender	10	0.3%
Something else	8	0.3%
Missing	26	0.9%
**Relationship Status**		
Single and not dating	652	21.6%
Single and dating or hanging out with someone	166	5.5%
In a relationship but not living together	215	7.1%
Living together but not married	309	10.2%
Married	1653	54.8%
In more than one relationship	16	0.5%
Missing	6	0.2%
**Age**		
18–29	590	19.6%
30–44	792	26.2%
45–59	789	26.2%
60+	846	28.0%
**Race and Ethnicity**		
White, non-Hispanic	1925	63.8%
Black, non-Hispanic	376	12.4%
Other Race, non-Hispanic	207	6.8%
Hispanic	468	15.5%
>2 Races, non-Hispanic	42	1.4%
**Education**		
Less than High School	301	10.0%
High School	815	27.0%
Some College	911	30.2%
Bachelor’s Degree or Higher	990	32.8%
**Household Income**		
Under $25,000	504	16.6%
$25,000–$49,999	624	20.7%
$50,000–$74,999	523	17.4%
$75,000–$99,999	412	13.6%
$100,000–$149,999	479	15.9%
$150,00 and over	475	15.7%
**Geographic Region of Residence**		
Northeast	531	17.6%
Midwest	634	21.0%
South	1150	38.1%
West	701	23.2%

### Prevalence of pleasure from anal touch

Approximately four out of ten women (43.5%, n = 1,283) reported experiencing pleasure with some form of anal touch (internal or external). One quarter (25.8%) of women had never tried any form of anal touch assessed. More specifically, 27.6% of respondents reported no prior experience with external anal touch, or “not knowing” how pleasurable that type of touch might be for them; 32.5% had never tried any penetrative/internal anal touch techniques or did not know how pleasurable they might be.

### Prevalence of pleasure during anal surfacing

As shown in Table 2, 40.3% of U.S. women have used and found pleasurable Anal Surfacing, or touch on the outside of the anus. One-third (34.0%) of women have found anal surfacing using a *partner’s* finger pleasurable whereas about one-fifth (20.3%) have found using their *own* finger pleasurable. Anal surfacing using a partner’s penis was reported as pleasurable by about one-third of women (31.4%) and about one-fifth (19.4%) reported using a sex toy for this kind of touch as pleasurable. About a quarter of participants noted they did not enjoy any form of anal surfacing (22.3%) and one-third of women reported they found no pleasure in each specific anal surfacing technique (31.6%–38.5%).

**Table 2:** Prevalence of anal surfacing techniques women find pleasurable (N = 2937, weighted).

**Any form of anal surfacing**	**Percentage of women who have used and found *any* technique pleasurable (n)**	**Percentage of women who have used and found *every* technique unpleasurable (n)**
	40.3% (1188)	22.3% (656)
**Sub-forms of anal surfacing**:	**Percentage of women who have used this technique and found it pleasurable (n)**	**Percentage of women who have used this technique and found it unpleasurable (n)**
Anal surfacing with partner’s finger	34.0% (997)	31.6% (924)
Anal surfacing with own finger	20.3% (596)	38.5% (1131)
Anal surfacing with a penis	31.4% (923)	34.0% (998)

### Prevalence of pleasure during anal penetration and, specifically, anal shallowing

As shown in Table 3, 34.6% of U.S. women have used and found pleasurable touch inside of the anus. Anal penetration using a *partner’s* finger was the most prevalent internal touch technique, found pleasurable by about over one-quarter (28.3%) of women. Half that number of women have found using their *own* finger pleasurable (15.4%). Anal penetration with a partner’s penis was reported as pleasurable by nearly the same proportion (25.7%) of women who enjoyed touch by a partner’s finger. Approximately one-sixth of women (17.4%) reported using a sex toy for this kind of touch as pleasurable. About a quarter of participants noted they did not enjoy any form of touch inside the anus (23.6%) and one-third of women reported they found no pleasure in each specific touch technique (30.9%–36.0%).

**Table 3:** Prevalence of anal penetration (either shallow or deeper) techniques women find pleasurable (N = 2931, weighted).

**Any form of anal penetration**	**Percentage of women who have used and found *any* technique pleasurable (n)**	**Percentage of women who have used and found *every* technique unpleasurable (n)**
	34.6% (1015)	23.6% (693)
**Sub-forms of anal penetration**:	**Percentage of women who have used this technique and found it pleasurable (n)**	**Percentage of women who have used this technique and found it unpleasurable (n)**
Anal penetration with partner’s finger	28.3% (828)	31.6% (926)
Anal penetration with own finger	15.4% (452)	36.0% (1054)
Anal penetration with a penis	25.7% (752)	34.0% (996)
Anal penetration with sex toy	17.4% (511)	30.9% (905)

Table 4 demonstrates that 38.2% of women who had experienced pleasure from any form of anal penetration found shallower depths of Anal Shallowing pleasurable, whereas 41.6% found deeper penetration pleasurable. Out of possible penetration depths, Anal Shallowing just barely inside of the anus such as with a fingertip was reported as pleasurable by the second greatest proportion of women; one-quarter (24.7%) of those who had experienced pleasure from any form of anal penetration enjoyed this shallow depth of penetration. The slightly deeper (‘about one knuckle inside’) sub-form of Anal Shallowing we assessed was found pleasurable by 13.5% of women who had experienced pleasure from any form of anal penetration. About a quarter found a finger’s depth pleasurable and 12.9% enjoyed depths more than a finger can typically reach.

**Table 4:** Depths of internal anal touch found most pleasurable by women endorsing pleasure from any form of anal penetration (N = 1015, weighted)^a^.

**Depths of internal anal touch**	**Percentage of women who have found this depth of anal touch pleasurable (n)**
Any form of anal shallowing	38.2% (388)
**Sub-forms of anal shallowing**:	
Just barely inside (such as the very tip of a finger)	24.7% (251)
About one knuckle inside	13.5% (137)
Any form of deeper penetration	41.6%
**Sub-forms of deeper anal penetration**:	
As deep as an entire finger can go	28.7% (291)
Deeper inside than an entire finger can usually reach	12.9% (131)
I don’t know	19.2% (195)

^a^11 women with missing responses to this item were excluded from these analyses (n = 1004)

### Anal pairing and types of pleasure experienced from anal touch

Women (N = 1,283) who reported pleasure with anal surfacing and/or anal penetration (shallow or deeper) were subsequently asked about the ways in which anal touch was pleasurable for them, as summarized in Table 5. Over one-quarter of women (27.6%) enjoy anal touch because it makes their orgasms feel more intense. The most prevalent way in which anal touch was reported as pleasurable was as a complement to other forms of touch (e.g. clitoral touch and vaginal penetration) when done simultaneously; we refer to this technique as Anal Pairing. Four out of ten (39.9%) women endorsing pleasure from any form of anal touch reported that anal touch makes other sexual touch more pleasurable for them and one-sixth (16.7%) of women specifically noted it makes reaching orgasm easier during other kinds of sexual touch.

**Table 5:** Ways in which anal touch is pleasurable reported by women endorsing pleasure from any form of anal touch (N = 1283, weighted).

Types of pleasure	Percentage of women who endorsed this type of pleasure from anal touch (n)
It has its own unique sensation that I find pleasurable	38.9% (499)
It can make my orgasms feel more intense	27.6% (355)
It feels profoundly intimate and emotional	18.2% (233)
The pleasure feels fuller or ‘bigger’ than other kinds of sexual pleasure	12.5% (160)
I get a thrill from the feeling that anal play is taboo	11.6% (149)
I can have orgasms just from anal touch/stimulation	9.2% (118)
Other	6.0% (76)
**Anal Pairing**:	
When it happens at the same time as other kinds of touch (like vaginal sex or clitoral touching), it can make the experience more pleasurable	39.9% (512)
It can make it easier for me to have an orgasm during other kinds of touch	16.7% (214)

### Prevalent ways in which women discover anal touch is pleasurable for them

For many women who currently enjoy anal touch, finding pleasure with it was a gradual process. Indeed, 56.1% of women did not find Anal Surfacing to be pleasurable the first time they tried it, but rather enjoyed it more over time. Similarly, 67.7% of women who now enjoy penetrative anal touch did not find it pleasurable at first, but rather came to enjoy it over time. As shown in Table 6, we assessed seven patterns through which women make the initial, often gradual, discovery that anal touch is pleasurable for them. The most prevalent discovery patterns involved women’s partners. For almost half of women (44.6%), their first experience of pleasure from anal touch came as a result of a partner approaching it in a way that worked for them. Four out of ten (39.4%) women noted the importance of the emotional connection they felt with the partner with whom they were trying anal touch as a critical factor in their discovery of anal pleasure. One-quarter of women suggested that physical factors such as having enough time to become aroused beforehand (24.7%) or simply using sufficient lubricant (amount self-determined; 22.7% of women) made the biggest difference in their discovery that anal touch could feel good. To a lesser extent, women (14.0%) noted that their own self-touch during masturbation was how they discovered anal touch could be pleasurable. There was a wide range of ages (14–72 years-old) at which women discovered anal touch was pleasurable for them. The mean age of discovery, however, was the same for both internal (n = 685) and external (n = 734) anal touch: 27 years-old.

**Table 6:** Prevalence of patterns in how women first discover that anal touch is pleasurable reported by women endorsing pleasure from any form of anal penetration (N = 1283, weighted).

Discovery patterns	Percentage of women reporting this discovery pattern (n)
A partner approached anal sex in a way that worked for me	44.6% (572)
Trying it with someone I loved or deeply cared for	39.4% (506)
I had enough "warm up time" beforehand	24.7% (317)
A partner just barely touched or brushed by my anus/butthole and I enjoyed it	23.7% (304)
Once I/we used enough lubricant	22.7% (292)
Trying anal touch or anal sex on my own terms (e.g., communicating about the way I wanted to do it and having that respected)	18.5% (238)
During self-exploration/masturbation, I found a way of anal stimulation that I liked	14.0% (179)
I felt an increased desire for anal sex during or since pregnancy	2.2% (28)
I felt an increased desire for anal sex during or after menopause	1.7% (22)

## Discussion

Despite the anatomic pleasure potential [4] of the anus to promote to maximize women’s sexual enjoyment [2, 3], the specific ways in which women might engage the anus—by themselves or with partners—as a part of their sexual repertoire has remained largely unexamined in the current sexuality literature. To address this gap, we used data from the *second OMGYES Pleasure Report* to providing the first detailed, population level description of the of the distinct anal stimulation and penetration techniques women engage to enhance their sexual pleasure during both solo and partnered sex. Specifically, through inductive, qualitative research followed by this nationally representative survey, we identified and named three previously undefined anal touch techniques that are found pleasurable by large proportions of U.S. women: Anal Shallowing, Anal Surfacing, and Anal Pairing.

Describing the ways in which women engage the anus in personally enjoyable ways is an important expansion of the existing anus-focused literature in several ways. One important contribution of this work is our naming of touch and stimulation techniques in lay phrasing that is usable by women and their partners. As we have suggested in our work focused on vaginal stimulation and penetration [24], for women to be comfortable communicating what new techniques they want to try and/or what existing techniques they want to try differently, the words and descriptions they use to do so must be both straightforward and accessible to them. For the vast majority of anal sexual practices, however, with some exceptions around oral-anal sexual touch (specifically referred to as *analingus*, *rimming*, a *rim job* or *tossing the salad)* and deep anal penetration with a hand or arm (specifically referred to as *anal fisting*, *handballing* or *brachioproctic eroticism*) [31], there has been a lack of standalone, clear terminology. For example, the term *Anal Surfacing* we developed refers to touch on the outside of the anus, which 4 out of 10 women have discovered is pleasurable. The technique we named *Anal Shallowing* refers to touch just inside the anal opening, no deeper than a fingertip/knuckle, which was found pleasurable by 38.2% of women who enjoy any form of anal penetration. This study also identified anal touch paired with simultaneous vaginal and/or clitoral touch—referred to as *Anal Pairing*—can work synergistically with other forms of sexual touch as an enhancement of women’s pleasure and orgasm. Language for knowing how and for what kinds of anal touch to ask a partner may be especially important since most prevalent forms of touch reported as pleasurable for women were done by a partner’s finger or penis. The wide range of ages at which women discovered that external and/or penetrative anal touch was pleasurable to them illustrates that new pleasurable touch techniques can be discovered at any age and may encourage women to continue exploring their pleasure throughout the lifespan. Thus, there is a wider anal sexual repertoire that women enjoy in everyday life than has been named in scientific literature or that is often discussed openly in society.

In addition, we also explored the dimensionality of these techniques, identifying *what* is being used for touch or stimulation (e.g., own or partner’s finger, penis, or sex toy), *the manner in which* it is being used (e.g., on the surface vs. inside, or just barely inside vs. one knuckle in vs. deeper than a finger’s length inside), *why* they find it pleasurable (e.g. increased orgasm intensity vs. thrill vs. intimacy) and *how* they discovered it (e.g. partner introduction vs. self-exploration). This granularity supports a women-centered models of anal sexuality in which women and/or their partners are empowered to choose anal touch and stimulation technique(s) that meets their own personal pleasure needs. As suggested earlier in this paper, most of what is “known”—either through scientifically literature or through popular culture—about using the anus during sex typically focuses on the anus being penetrated by another body part [1, 5–15] or emphasizes the adverse outcomes (e.g. disease, pain or coercion) associated with anal sex for women [16–18]. Our detailed, nationally representative data on the prevalence of women’s participation in techniques and their variants, their technique discovery, and their motivation for techniques, is an important validation for women and/or their partners that individuals *like themselves* can and do actively choose anal touch and stimulation to meet their own personal pleasure needs. Information such as this is vital for women’s ability to “normalize” their participation in and motivations for a behavior that is often socially stigmatized [19].

## Limitations and strengths

Several limitations associated with the current data should be considered. From a measurement perspective, some survey items assessed technique participation in general, whereas others assessed technique participation in association with sexual pleasure, which could challenge disentangling a participant’s reported use of technique from their motivation for choosing that technique, as well as disentangling their actual experience of pleasure or displeasure. For example, regarding pleasure from deep penetration preference depths, we asked participants “What depth have you found anal stimulation most pleasurable?” A participant’s selection of option (e.g., “one knuckle or less”) is comparative in nature and does not necessarily mean they do not enjoy another option (e.g., “two knuckles or more”). It is possible this approach could result in an underestimate of enjoyment in these approaches. Future measurement approaches to address this challenge could include both assessing lifetime participation in specific techniques, with linking questions regarding motivation or expected pleasure, as well as actual pleasure outcomes for those who affirmed participation.

In addition, participants only included female-identified individuals; we queried men neither about their experiences with any of the anal techniques assessed here nor about how such participation may have impacted their own or their partner’s sexual pleasure. It will be important for ongoing studies to understand how selection and enjoyment of specific anal techniques are linked to partnership factors like relationship satisfaction and happiness, communication, and sexual satisfaction.

Finally, we did not assess any oral-anal behaviors as part of the larger survey informing this paper. While such behaviors are part of a larger anal sexual behavior repertoire for women [1, 13, 14], survey lengths precluded their inclusion. It will be important in future work for these items to be added, particularly for understanding how participation and pleasure compare to the touch and penetration behaviors we assess here. These limitations are balanced with several methodological and substantive strengths of this study. From a methodological perspective, our use of a nationally representative probability sample permits generalization of findings to the broader population of women in the United States. Other sampling approaches common in sexual and reproductive health research, including convenience, clinical or community-based recruitment, do not allow this level of comparison. In addition, our use of Ipsos’ KnowledgePanel^®^ affords several data collection advantages, including access to already experienced survey participants, secure survey storage and sending of participation reminders to potential respondents. Ipsos also controls the number of surveys sent to each member, minimizing the unit- and item-level missingness on any given survey. Another methodological strength is online data collection, which facilitates survey completion in a setting of the participant’s choosing, thereby increasing data confidentiality and participant comfort with answering questions about potentially sensitive topics, like sexual behavior and sexual pleasure.

## Conclusion

Data from this U.S. nationally representative survey provide descriptions of and prevalence estimates for three techniques women have discovered to make anal stimulation and penetration more pleasurable: Anal Surfacing, Anal Shallowing, and Anal Pairing. Our findings contribute to the growth of much needed, detailed literature on the ways in which women discover, engage in, and enjoy anal stimulation and penetration. Knowledge of these techniques can enable women to better identify their own preferences, communicate about them and advocate for their sexual pleasure.

## Supporting information

S1 Table Definitions for and sexually explicit line drawing illustrations of Anal Surfacing, Anal Shallowing, and Anal Pairing techniques for experiencing sexual pleasure during anal touch. (DOCX)
