# Supplementary material for: Women’s techniques for pleasure from anal touch: Results from a U.S. probability sample of women ages 18–93
Source: PLoS One. 2022 Jun 29;17(6):e0268785. doi: 10.1371/journal.pone.0268785 (PMC9242470; doi:10.1371/journal.pone.0268785)
Supplement: S1 Table — (DOCX) [file pone.0268785.s001.docx]

**Supplemental Table. Definitions for and sexually explicit line drawing illustrations of Anal Surfacing, Anal Shallowing, and Anal Pairing techniques for experiencing sexual pleasure during anal touch.**

| **Technique** | **Definition** | **Example Illustration** |
| --- | --- | --- |
| **Anal surfacing** | Sexual touch by a finger, penis, or sex toy on and around the anus | 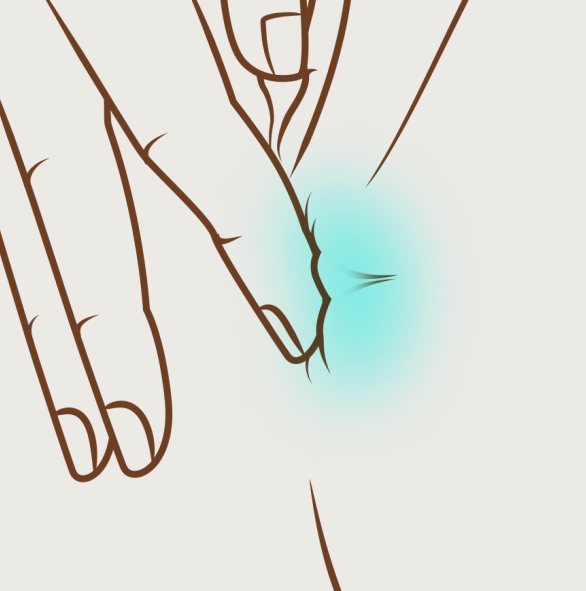 |
| **Anal shallowing** | Shallow penetrative touch by a finger, penis, or sex toy just inside the anal opening, no deeper than a fingertip/knuckle | 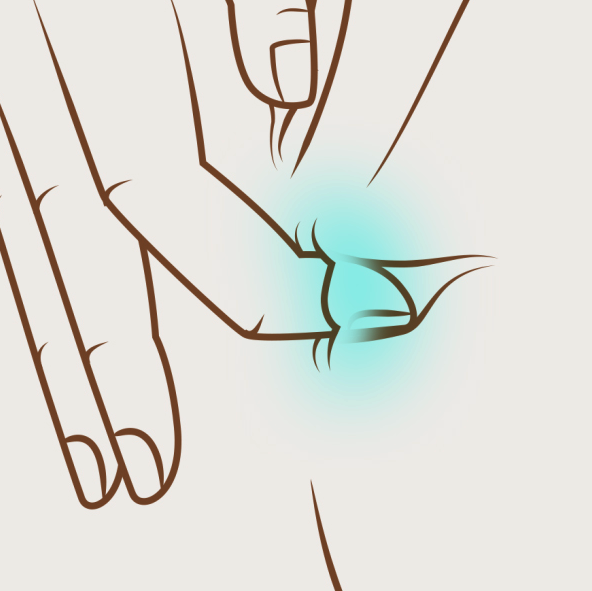 |
| **Anal pairing** | Sexual touch on or inside the anus that happens at the same time as other kinds of sexual touch such as vaginal penetration or clitoral touching | 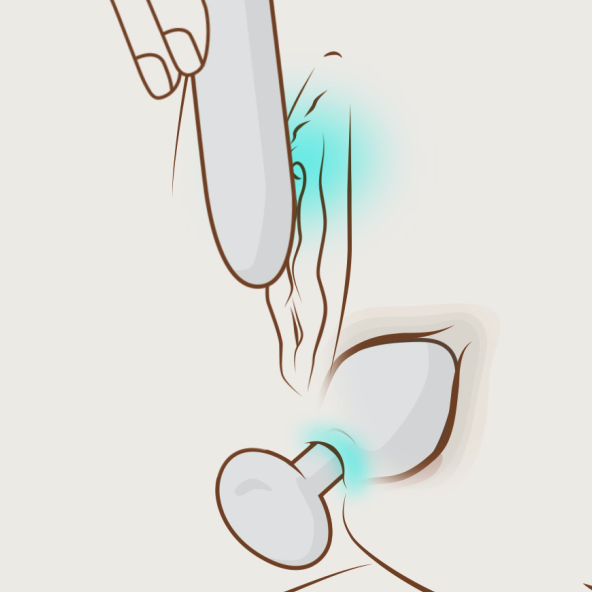 |
